# Supplementary material for: Dynamic transcriptomic profiles of zebrafish gills in response to zinc supplementation
Source: BMC Genomics. 2010 Oct 11;11:553. doi: 10.1186/1471-2164-11-553 (PMC3091702; doi:10.1186/1471-2164-11-553)
Supplement: Additional file 2 — Interactive Direct Interaction Network representing the molecular interactions between zinc, copper, iron, calcium and proteins encoded by transcripts changed by zinc supplementation. Mini web-site containing index.html and hyperlinked pages in subdirectory describing a Direct Interaction Network automatically generated based on curated interactions contained within the proprietary PathwayArchitect database. Ovals represent proteins and the circles symbolize metal ions. Objects are coloured by their abundance in zebrafish at the time-point they were significantly different from the control is a scale from -4 fold (dark green) to +4 fold (dark red). Where significant differences were found at more than one time-point, the colour overlay shows expression at the first instance. Dark blue squares denote 'binding', and light blue squares 'expression'; green squares stand for 'regulation', green diamonds for 'metabolism', and green circles for 'promoter binding'. Arrow heads indicate directionality of the interaction where annotated. All nodes and edges can be further interrogated by selecting the relative area of the image. [file 1471-2164-11-553-S2.zip › PathwayArchitect Zn xs DIN/888182.html]

# BINDING:

|  |  |
| --- | --- |
| Type | BINDING |
| Effect | None |


---

|  |  |
| --- | --- |
| Score | 0 |


---

|  |  |
| --- | --- |
| Reference Count | 6 |


---

|  |  |
| --- | --- |
| Mechanism | Unknown |


---

|  |  |
| --- | --- |
| Reference:0 || Sentence | "It will be seen that control of the actin-myosin reaction is not only through Ca(2+)-binding to TnC, but also through steric, cooperative and allosteric processes involving all of the main myofilament proteins-actin, myosin, tropomyosin (Tm), troponin T (TnT), TnC, and TnI." |
| PMID | 8540387 |
| Year | 1995 |
| Species | Human |
| Journal | Adv Exp Med Biol |
| RefScore | 1 |
| Source | PArchNLP |
  |
|  |  |


---

|  |  |
| --- | --- |
 Reference:1 || Sentence | "The regulatory protein troponin (Tn) located on actin filament consists of three subunits: TnT--binds troponin to tropomyosin, TnC--binds divalent calcium ions, and TnI--affects myosin-actin interactions." |
| PMID | 10638675 |
| Year | 1999 |
| Species | Human |
| Journal | Physiol Res |
| RefScore | 2 |
| Source | PArchNLP |
  ||  |  |


---

|  |  |
| --- | --- |
 Reference:2 || Sentence | "Cardiac muscle activation is initiated by the binding of Ca(2+) to the single N-domain regulatory site of cardiac muscle troponin C (cTnC)." |
| PMID | 12511564 |
| Year | 2003 |
| Journal | J Biol Chem |
| RefScore | 0 |
| Source | PArchNLP |
  ||  |  |


---

|  |  |
| --- | --- |
 Reference:3 || Sentence | "The distance between Ca2+-binding site III in the C-terminal domain and Cys35 in the N-terminal domain in cardiac muscle troponin C (cTnC) was determined with a single-tryptophan mutant using bound Tb3+ as the energy donor and iodoacetamidotetramethylrhodamine linked to the cysteine residue as energy acceptor." |
| PMID | 10716180 |
| Year | 2000 |
| Species | Rat |
| Journal | Protein Sci |
| RefScore | 0 |
| Source | PArchNLP |
  ||  |  |


---

|  |  |
| --- | --- |
 Reference:4 || Sentence | "Arguments are further presented in support of the concept that Ca2+, in conjunction with troponin C, is the main determinant of cardiac contractility and that dT/dt reflects a dynamic equilibrium between free and troponin-bound Ca2+." |
| PMID | 8076447 |
| Year | 1994 |
| Species | Human |
| Journal | Comp Biochem Physiol Physiol |
| RefScore | 2 |
| Source | PArchNLP |
  ||  |  |


---

|  |  |
| --- | --- |
 Reference:5 || Sentence | "Physiological studies suggest the following. 1) Ca(2+) binding to Tn/Tm exposes sites on actin to which myosin can bind. 2) Ca(2+) regulates the strong binding of M.ADP.P(i) to actin, which precedes the production of force (and/or shortening) and release of hydrolysis products. 3) The initial rate of force development depends mostly on the extent of Ca(2+) activation of the thin filament and myosin kinetic properties but depends little on the initial force level. 4) A small number of strongly attached cross bridges within an A(7)TmTn regulatory unit can activate the actins in one unit and perhaps those in neighboring units." |
| PMID | 10747208 |
| Year | 2000 |
| Species | Human |
| Journal | Physiol Rev |
| RefScore | 3 |
| Source | PArchNLP |
  |


---

|  |  |
| --- | --- |
